# The Effect of Unconventional Cytokine Combinations on NK-Cell Responses to Viral Infection

**DOI:** 10.3389/fimmu.2021.645850

**Published:** 2021-03-19

**Authors:** David E. Ochayon, Stephen N. Waggoner

**Affiliations:** ^1^ Center for Autoimmune Genomics and Etiology, Cincinnati Children’s Hospital Medical Center, Cincinnati, OH, United States; ^2^ Department of Pediatrics, University of Cincinnati College of Medicine, Cincinnati, OH, United States

**Keywords:** innate lymphoid cell, NK cell, interleukin, transforming growth factor, interferon gamma

## Abstract

Cytokines are soluble and membrane-bound factors that dictate immune responses. Dogmatically, cytokines are divided into families that promote type 1 cell-mediated immune responses (e.g., IL-12) or type 2 humoral responses (e.g., IL-4), each capable of antagonizing the opposing family of cytokines. The discovery of additional families of cytokines (e.g., IL-17) has added complexity to this model, but it was the realization that immune responses frequently comprise mixtures of different types of cytokines that dismantled this black-and-white paradigm. In some cases, one type of response may dominate these mixed milieus in disease pathogenesis and thereby present a clear therapeutic target. Alternatively, synergistic or blended cytokine responses may obfuscate the origins of disease and perplex clinical decision making. Most immune cells express receptors for many types of cytokines and can mediate a myriad of functions important for tolerance, immunity, tissue damage, and repair. In this review, we will describe the unconventional effects of a variety of cytokines on the activity of a prototypical type 1 effector, the natural killer (NK) cell, and discuss how this may impact the contributions of these cells to health and disease.

## Introduction

Cytokines serve as messengers of the immune system, dictating the functional differentiation of immune cells to suit the nature of threats to health (1). The dichotomy between cell-mediated responses against intracellular viruses or bacteria and the need for humoral responses against parasitic infection is dictated by the cytokine milieu (2). Within these dual arms of the immune response, cytokines fine tune the immune response, for example, by determining isotype class switching (3). The capacity of innate immune cells to recruit effector cells, process and present antigens, trigger tolerogenic responses, and promote tissue repair are all determined by and executed *via* release of cytokines (4, 5).

The orthodox dogma of cytokine responses suggests a dichotomy between type 1 and type 2 cytokines, meaning that type 1 responses will counteract type 2 responses and vice versa (6, 7). While the prototypical type 1 cytokine IL-12 promotes IFN-γ expression, the type 2 cytokine IL-4 can suppress this response (7). Nevertheless, IL-4 can paradoxically promote IFN-γ expression and memory responses in CD8 cytotoxic T and NK cells (8, 9). In this review, we aim to elucidate some of the complexity of cytokine signaling and its function during viral infection.

Natural killer (NK) cells are part of the innate immune compartment with a fundamental role in combating viral infections and eliminating tumor cells (10). NK cells kill targets rapidly *via* release of perforin- and granzyme-containing cytolytic granules, or more slowly *via* death receptor (e.g. FasL) interactions with target cells. At various points during the immune response, NK cells are vital sources of cytokines (11). These include the hallmark cytokine interferon-gamma (IFN-γ) as well as pro-inflammatory mediators like tumor necrosis factor alpha (TNF-α) and granulocyte-macrophage colony-stimulating factor (GM-CSF) (12, 13). There is also evidence that NK cells can produce type 2 (e.g. IL-5, IL-13) and immunoregulatory cytokines (e.g. IL-10, TGF-β). This variety of functional contributions of NK cells is likely dictated by the cytokine milieu to which they are exposed during an immune response. How cells translate signals from this complex cytokine milieu into concerted functional activity remains incompletely defined. The following review aims to shed light on NK-cell responses to unconventional cytokines.

## The IL-12 Family of Cytokines

NK cells are highly responsive to IL-12 which triggers IFN-γ production *via* STAT4 phosphorylation and Tbet transcriptional activity (14, 15). Bioactive IL-12 is heterodimer of the IL-12p40 and IL-12p35 subunits. The role of IL-12 in NK-cell biology has been extensively reviewed elsewhere (11), and will not be discussed in detail in this review.

IL-23 is a closely related IL-12 family member; it is a heterodimer of IL-12p40 and IL-12p19 subunits. IL-23 is released from activated myeloid cells, including dendritic cells and macrophages, which are distributed in peripheral tissues such as skin, lung and intestine (16). IL-23 shares a resemblance with IL-12 in its ability to induce the release of IFN-γ from NK cells. In addition to phosphorylation of STAT4, IL-23 also triggers phosphorylation of STAT3 (16). However, in mucosal tissues IL-23 mediates a wide variety of immune responses (17). In T cells, it supports the proliferation and activation of Th17 cells, which express IL-17 and IL-22 (18). IL-23 plays a similar role in triggering production of these cytokines by group 3 innate lymphoid cells (ILC3) (19).

IL-17 and IL-22 play key roles in mucosal immunology (16).

**Figure 1:**
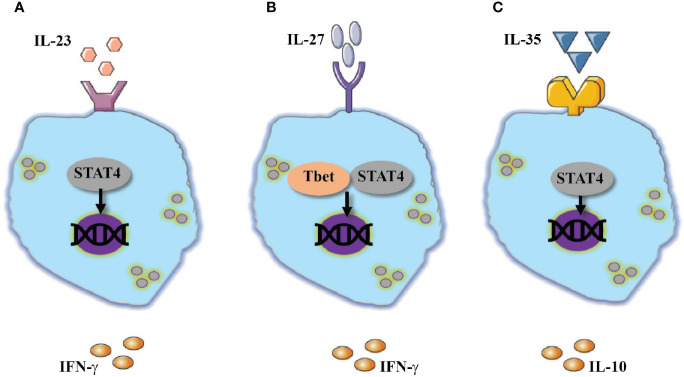
Additional members of IL-12 family cytokines modulate NK-cell responses. Activation of NK cells are mediated by: **(A)** IL-23 or **(B)** IL-27 through activation of STAT4 and Tbet which resulted in IFN-γ expression. **(C)** An opposite effect to the above-mentioned cytokines was documented by IL-35 which also utilize STA4 and promote the expression of IL-10.

In humans, CD56^bright^ NK cells exhibit increased expression of IL-23R in comparison to CD56^dim^ NK cells. Consistent with this expression, CD56^bright^ compared to CD56^dim^ NK cells showed superior ability to express IFN-γ in response to IL-23 stimulation (20). In addition, IL-23 is critical during *T. gondii* infection for promoting NK cell activation and expression of IFN-γ (**Figure 1A**). With the growing appreciation of IL-23 role in mucosal and autoimmune pathologies (21, 22), additional studies are required to fully understand the role of IL-23 in NK-cell responses.

Interleukin-27 (IL-27) is a heterodimeric cytokine in the IL-12 family comprised of Epstein-Barr virus–induced gene 3 (EBI3) and IL-27p28. The IL-27 signal is transduced by its receptor which contains gp130 and WSX1 subunits (23). Macrophages, microglia, dendritic cells, and inflammatory monocytes are the main sources of IL-27. Endothelial and epithelial cells also express IL-27 (23). The role of IL-27 in T cell biology is diverse; initial studies showed that IL-27 mediates the expression of Tbet, STAT4 and IFN-γ in naïve T cells (23, 24). However, recent studies have demonstrated that IL-27 can also suppress Th1- inflammatory responses by promoting the survival and proliferation of T regulatory cells and enhancing their ability to express IL-10 (25, 26). In a similar fashion to its effect on Th1 cells, IL-27 is suggested to enhance the inflammatory responses of NK cells. Indeed, human NK cells stimulated with IL-27 showed diverse cytokine release which included increased release of IL-10 and IFN-γ (27). Moreover, IL-27 stimulated NK cells were shown to have increased cytolytic function associated with elevated expression of NKp46 and Tbet (28). Combined Stimulation of NK cells with IL-27 in combination with IL-18 or IL-15 enhanced the effects of IL-27 on NK cell activity (28, 29). *In vivo*, IL-27 mediates early NK cells responses during viral infection (30, 31). In an experimental model of influenza, deletion of IL-27R or EBI3 resulted in reduced expression of IFN-γ and repressed degranulation responses by NK cells (31). The reduced response of NK cells was linked to increased susceptibility to viral infection. Interestingly and different from human NK cells, *in vitro* stimulation of mouse NK cells with added IL-27 did not enhance IFN-γ expression in IL-12 and IL-18 stimulated NK cells (31). Nevertheless, IL-27 increased the expression of IFN-γ when murine NK cells were stimulated *via* NKG2D (31).

In addition to these reported stimulatory effects, IL-27 can also suppress NK-cell inflammatory responses *via* an indirect mechanism. The interaction of human NK cells with IL-27-stimulated monocytes resulted in reduced expression of IFN-γ but did not alter NK-cell cytotoxic responses (32). The inhibition of IFN-γ release was attributed to increased expression of human leukocyte antigen class I histocompatibility antigen, alpha chain E (HLA-E), which was demonstrated to directly interact with NK cells (33).

The above-mentioned studies demonstrate that on one hand, IL-27 possesses a similar function as IL-12 and can promote NK cells activation and assist in anti-microbial responses (**Figure 1B**). On the other hand, indirectly, IL-27 can downregulate NK-cell responses. Thus, additional studies should clarify under which circumstances does IL-27 activate or suppress NK-cell responses.

IL-35 is another member of the IL-12 family, with structural similarity to IL-27 and IL-23. IL-35 is a heterodimeric cytokine comprised of the IL-12p35 and EBI3 subunits (34). Several T-cell subsets, but most prominently regulatory T cells, express IL-35 (35), which is suggested to suppress inflammatory responses in a variety of cells (34).

In response to various stimuli, mouse NK cells were demonstrated to express EBI3 and IL-35, but not p28, an IL-27 subunit (36). A similar phenomenon was observed in the experimental model of murine cytomegalovirus (MCMV). Post infection NK cells were shown to express EBI3 and subunit p35 but not p28 which is associated with IL-27. Moreover, NK cells derived from EBI3-deficient mice, expressed a lesser amount of IL-10 and showed reduced viral latency (36). Although IL-35 appears to be an immunosuppressive agent, reduced activation of cytotoxic cells such as NK cells and CD8 T cells might be beneficial to the host by limiting tissue injury (37, 38) (**Figure 1C**).

## IL-1 Family of Cytokines

IL-18 is a member of the type 1 cytokine family, formerly known as interferon-γ-inducing factor. Indeed, the combination of IL-18 with IL-12 provokes strong IFN-γ expression in both human and murine NK cells (13). IL-18 activates the NF-κB and p38 MAPK pathways, and the latter enhances the stability of IFN-γ transcripts (39, 40). The combined stimulation of IL-18 and IL-12 also mediates release of additional cytokines such as GM-CSF and TNF-α (13). The role of IL-18 in anti-viral responses *in vivo* includes support for NK cell expansion and activation (41–44).

IL-33 is one of the most recently discovered members of the IL-1 family (45, 46). Previous studies showed that IL-33 has dual functionality. Intracellularly, IL-33 was demonstrated to interact with chromatin with unknown consequences for cellular biology (47). Extracellularly, IL-33 promotes inflammatory responses as a damage associated molecule, released passively from cells after injury (45). Early studies showed the ability of IL-33 to mediate type 2 responses in basophils, T cells, mast cells and ILC2 cells (45). However, IL-33 also mediated type 1 responses in pre-stimulated macrophages, which resulted in increased expression of TNF-α and IL-1β (48–50). During viral infection, IL-33 is vital for Th1 responses and infection resolution (51). In mouse NK cells, IL-33 enhanced the release of IFN-γ in IL-12 stimulated NK cells (52). In addition, in an experimental model which mimics the injurious effect of smoking damage, IL-33 is released from lung tissue and couples with inflammatory signals caused by influenza virus infection to provoke greater IFN-γ expression than seen in the absence of smoke (53). In addition to its direct effects on NK cells, IL-33 can also enhance the expression of IL-12p40 and thereby indirectly enhance IFN-γ production by NK cells (53). Recent reports showed that IFN-γover-production mediates airway hypersensitivity (AHR) and inflammation which is not affiliated with classic AHR inducers such as neutrophils (54). Smokers and patients with chronic obstructive pulmonary disorder (COPD) or severe asthma exhibit increased susceptibility to respiratory viral infections such as influenza virus, respiratory syncytial virus, and rhinovirus (55–60).

**Figure 2:**
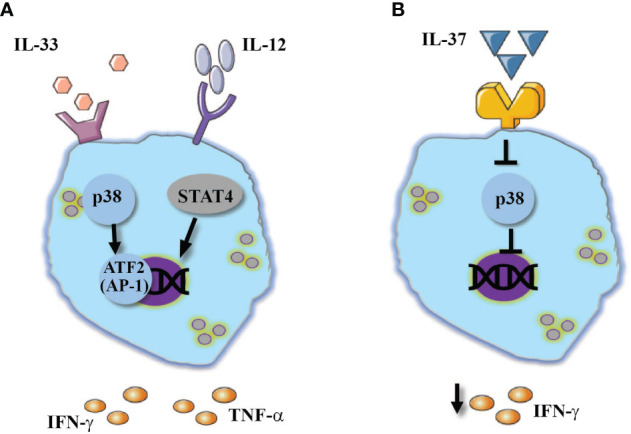
IL-1 family cytokines regulate NK-cell responses. The combined stimulation of IL-12 stimulated NK cell with: **(A)** IL-33 mediates the activation of p38 pathway which resulted in enhanced IFN-γ and TNF-α expression. **(B)** In contrast, IL-37 was shown to inhibit p38 MAPK signaling pathway which resulted in reduced IFN-γ expression.

Human NK cells showed a similar response to IL-33 (**Figure 2A**). IL-33 did mediate the release of IFN-γ, however, when combined with IL-12 or IL-23 it mediated significant increase of IFN-γ expression (61). In a similar fashion to IL-18, IL-33 combined with IL-12 mediated the release of inflammatory cytokines such as TNF-α and GM-CSF (62). Mechanistically, IL-33 enhanced the expression of NK-cell inflammatory cytokines *via* p38 MAPK signaling pathway and stabilization of *IFNG* transcripts (52, 62). IL-33 also induced activation of a disintegrin and metalloproteinase (ADAM)-17, which enhances the release of TNF-α from NK cells (62).

Of note, IL-33 was suggested to have a similar role to IL-18 in the context of modulating NK-cell memory responses. In an experimental model of MCMV infection, an increase in the expression of IL-33 was detected three days after infection (63). Furthermore, the IL-33 receptor ST2 (*Il1rl1*) was essential for proliferation of Ly49H+ memory NK cells and their ability to eliminate virus-infected cells (63).

In a similar fashion to IL-18 and IL-33, IL-1 also enhances IFN-γ expression in IL-12 stimulated NK cells (64). In humans, CD56^bright^ NK cells exhibit greater sensitivity to IL-1β than CD56^dim^ NK cells (65). Earlier studies showed that IL-1β can stimulate NK cell cytotoxicity (66).

In contrast to the rest of the IL-1 family, the newly discovered IL-37 predominately suppresses innate inflammatory responses (67–69). In hepatocellular carcinoma patients, increased expression of IL-37 within the tumor environment was resulted in increased infiltration of CD57^+^ NK cells into the tumor as well as a better prognosis (70). A possible explanation of improved NK cell function in the presence of IL-37 could be drawn from Qi et al. (2018). In an experimental model of influenza virus infection, anti-viral treatment combined with IL-37 increased the expression of IL-18 receptor (71), where IL-18 can promote NK-cell cytotoxic responses which in turn contribute to viral clearance (12). Moreover, IL-37 suppresses p38 MAPK signaling pathway (71), which was demonstrated to activate ADAM17 (72). In human NK cells, ADAM17 was demonstrated to shed CD16 from cell membrane (73), thus we can speculate that IL-37 diversely downregulates ADAM17 activation and TNF-α release from NK cells, while assisting in the preservation of CD16 expression, a molecule that plays a key role in ADCC. Thus, it appears that although IL-37 is characterized as an anti-inflammatory cytokine, it promotes NK cells functionality in various pathologies (**Figure 2B**).

## Interferons and IL-15

IL-15 is a common-γ chain cytokine which, unlike other cytokines, can activate NK cells as a trans- or cis-presented membrane bound cytokine (74). IL-15 plays a pivotal role in NK-cell biology (1). The interaction of IL-15 with its receptor *via* STAT5 phosphorylation mediates the proliferation, differentiation, survival, and activation of NK cells (75). Although IL-15 can activate NK cells on its own, the combination of IL-15 with cytokines such as IL-12, IL-18 or their combination was shown to increase the repertoire of released cytokines and was also demonstrated to mediate cytokine induced memory NK-cell responses (76).

In the context of viral infection, IL-15 is a key mediator in NK cell control of viral replication *via* cytotoxicity (77). Interestingly, IL-15 serum levels were shown to be associated with pediatric viral bronchiolitis severity, which might indicate that NK cells activated by IL-15 could play a harmful role in the course of viral infection (77).

In a similar fashion to IL-15, type I interferons IFN-α and IFN-β also play key roles in NK cell anti-viral responses. In the absence of type I interferons receptor or its key signaling molecule STAT1, NK cells showed impairment in survival, proliferation and reduced capacity to clear virally infected cells (78). Nonetheless, continuous stimulation with IFN-α or with IL-15 were shown to promote NK-cell exhaustion which resulted in reduced IFN-γ expression and cytotoxicity (78–80).

The IFN family also includes IL-28a/IL-28a and IL-29. The immune responses of these type III interferons are similar to those induced by type I interferons (81). These cytokines signal *via* IL-28R and IL-10R2 (81). In regard to NK cell responses, type III interferons were shown to activate NK-cell responses in the course of influenza infection and bacterial infection model (82). In the absence of IL-28R, a reduced response of NK cells was detected (83).

## Type 2 Cytokines

IL-4 is a common-γ chain cytokine, and it serves as a central cytokine in shaping type 2 responses (84–86). In T cells, it was shown to mediate the differentiation of Naïve T cells to Th2 *via* signaling activation signal (STAT) 6 and GATA3 (87, 88). In response to IL-4, Th2 cells release additional type 2 cytokines such as IL-13, IL-5 and IL-4 (89, 90). Similarly, IL-4 was shown to attract and activate ILC 2 which resulted in additional release and expression of type 2 cytokines (91). Thus, from the orthodox perspective, IL-4 appears to strictly activate Th2 related responses important in humoral immunity. Yet, IL-4 is also vital for the development of memory CD8 T cells (92, 93) and for enhanced expression of IFN-γ (94).

*In vivo*, mouse NK cells respond to IL-4 administration with an increased expression of IFN-γ several hours after IL-4 administration. The enhanced expression of IFN-γ levels was related to the expression of STAT6 but not STAT4 (9). In a different study, overexpression of IL-4 by hydrodynamic injection primed splenic and liver NK cells to respond to subsequent stimulation with IL-12 and IL-21, resulting in increased expression of IFN-γ, IL-10 and GMCSF. In addition, NK cells derived from mice that overexpressed IL-4 showed increased levels of granzyme B and elevated cytotoxicity towards YAC-1 cells. Interestingly, overexpression of IL-13, which share similar responses with IL-4, in a similar experimental setting did not show similar results that were induced by IL-4 (95).

In a similar manner to type 1 cytokines, IL-4 was shown to promote increased expression of IFN-γ when it was combined with type 1 cytokines such as IL-12 and IL-2 (96). In this context, the IFN-γ stimulating capacity of IL-4 was independent of STAT6. The ability of IL-4 to support the activation of STAT5 phosphorylation likely mediates IFN-γ expression (97).

**Figure 3:**
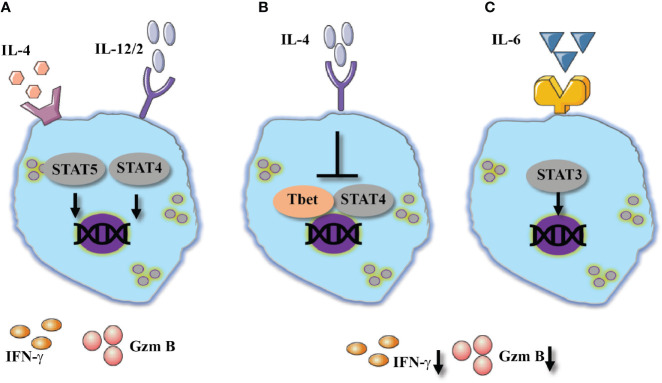
Type 2 cytokines show diverse effect of NK cells activation. Two outcomes in response to IL-4, **(A)** Alone or combines with 1 cytokines IL-4 mediates NK cells activation which resulted in increased expression of IFN-γ, potentially via STAT5 and STAT4. **(B)** Suppressive effect of IL-4 resulted in reduced IFN-γ and granzyme B expression suggested by the inhibition of Tbet and STAT4 signaling pathway. **(C)** IL-6 shows similar suppressive effect via STAT3 signaling pathway.

In contrast to the aforementioned effects of IL-4 as a NK-cell stimulant, pretreatment of human NK cells with IL-4 prior to stimulation *via* NKp46 did not enhance cytokine expression (98). Yet, IL-4 stimulation did dampen cytolytic responses of NK cells against targets lacking MHC expression. Lastly, pretreatment of human NK cells with IL-4 prevented their ability to interact with vascular endothelium; this phenomenon is suggested to limit the ability to of NK cells to recruit immune cells upon interaction with endothelial cells (99). The effects of IL-4 on NK cell responses are summarized in **Figures 3A**, **B**.

Of note, a study compared the effect of IL-13 versus IL-4 stimulation on human NK cells. Cells that were stimulated in the presence of IL-13 in comparison to IL-4, showed increased released of IFN-γ (100). No similar effect was detected in model animal, however, the above-mentioned study raised the question whether IL-4 and IL-13 activate similar signaling pathways, since both of these cytokines show a redundant role in pathologies such as asthma and atopic dermatitis.

IL-6 is a pluripotent cytokine that contributes to transcriptional programs for differentiation of regulatory, follicular and IL-17-producing subsets of CD4 T cells (101). Moreover, IL-6 activates a variety of inflammatory responses in hematopoietic cells (102). IL-6 was shown to downregulate IFN-γ expression and cytotoxic responses in CD8 T and NK cells (103, 104). Specifically, human NK cells stimulated in the presence of IL-6 were shown to have reduced expression of perforin and granzyme B, which resulted in reduced cytotoxicity (104). Validation to these findings was observed during the recent COVID-19 pandemic, in which NK cells derived from severe COVID-19 patients, compared to healthy patients showed reduced expression of granzyme A that was associated with IL-6 serum levels, in addition to reduced perforin expression (105).

Mechanistically, IL-6 mediates the phosphorylation of JAK3/STAT3 and the upregulation of suppressor of cytokine signaling 3 (SOCS3) (103). In CD8 T cells, SOCS3 activation was shown to downregulate STAT4 phosphorylation which mediates effector functions (94). Moreover, IL-6 presence was shown to mediate programmed death-ligand (PD-L)1 expression on NK cells, a molecule which is associated with reduced cytotoxicity of NK cells (106). Inhibition of JAK3 or IL-6 blockade (tocilizumab) revoked the suppressive effect of IL-6 and improved NK-cell cytotoxicity. Understanding the kinetics of IL-6 and its full effect of NK cells will provide additional lines of treatment in acute life-threatening viral infections (**Figure 3C**).

## Immunoregulatory Cytokines

### IL-10 and TGF-β

Transforming growth factor beta (TGF-β) is a central cytokine in T cell biology. In combination with IL-6, TGF-β activates the transcriptional program for T regulatory and Th17 cells (107). Studies aimed to understand the role of TGF-β in ILC biology showed that TGF-β is also essential for intestinal ILC3 fate and function (108). The interaction of TGF-β with its receptor mediates its signal to mothers against decapentaplegic homolog (SMAD)2 and SMAD3 which in turn promote SMAD4 translocation into the nucleus (109). In the context of cancer immunology, TGF-β was shown to downregulate NK-cell cytotoxic responses in addition to its ability to mediate the release of tumor supporting cytokines such as vascular endothelial growth factor (VEGF)-A (110, 111). Similar results were depicted in human NK cells study, in which the addition of TGF-β to IL-2/15 stimulated NK cells resulted in reduced IFN-γ, granzyme B and CD107a expression, all of which were attributed to the metabolic changes that TGF-β initiates in NK cells. All those effects were reversed when TGF-β receptor was inhibited or knocked down (111, 112).

NK cells are also known to be activated by antibody-dependent cell-mediated cytotoxicity (ADCC) *via* the interaction of NK cells with antibody-coated target *via* CD16 (113). In a study performed by Trotta et al., TGF-β was shown to downregulate NK cell activation mediated by CD16 ligation (114). The authors suggested that the inhibitory effect of NK-cell ADCC responses is mediated by SMAD3 which is downstream to TGF-β interaction with its receptor (114).

Thus, it appears that TGF-β possesses the potential of downregulating NK-cell responses. Indeed, high levels of TGF-β were shown in chronic Hepatitis B (HBV) patients. Sun et al. showed NK cells dysfunction and reduced activation markers such as NKG2D and 2B4 were negatively associated with TGF-β levels in HBV patients. The blockade of TGF-β restored the inhibitory effect of TGF-β on NK-cell activation markers and responses (115). TGF-β was shown to protect the host survival in the presence of lymphocytic choriomeningitis virus (LCMV) infection by modulating CD8 T cell responses (116). In the specific case of HBV, although NK cells were shown to assist in viral clearance, they are also suggested to mediate liver injury (117). In this regard, high expression level of TGF-β was reported in COVID-19 patients, and thus it is enticing to examine whether those high levels of TGF-β are harmful or protective. Additional studies will be needed to determine the full spectrum of TGF-β in the context of viral responses.

Interestingly, an unorthodox role of SMAD4 was shown in activating the NK-cell transcriptional program. Specific deletion of SMAD4 (*Smad4* ^f/f^Ncr1*^iCre^*) resulted in increased frequency of ILC1 at the expense of NK cells. Moreover, *Smad4* ^f/f^Ncr1*^iCre^* compared to *Smad4* ^f/f^ mice showed reduced ability to clear tumor cells and clear virally infected cells (114). The authors associated the important role of SMAD4 in NK cells to TGF-β signaling which was not dependent on TGF-β receptor (114, 118).

A study led by Wang et al. showed that SMAD4 is critical for NK cells homeostasis and maturation, however, the crucial effect of SMAD4 was independent to the role of TGF-β receptor 2 (119). The authors suggested that SMAD4 is mediating its non-canonical functions in cooperation with JunB, as both SMAD4 and JunB were shown to be in interaction with the promoter region of granzyme B (GZMB), which mediates NK cells cytolytic responses (119).

Another cytokine with inhibitory effects is the pleiotropic cytokine IL-10. Numerous studies documented the suppressive effect of IL-10 on inflammatory responses (120). In animal models, IL-10 was shown to modulate T cell responses and its deficiency was resulted in dysregulated T cell responses which led to autoimmunity and intestinal inflammation, demonstrating its critical role in T cell biology (121, 122). On the other hand, IL-10 was shown to moderate harmful inflammatory responses (123). In LCMV infected mice, IL-10 was demonstrated to downregulate the excessive innate and adaptive proinflammatory responses, and thus provides tissue protection at a cost of viral persistence (124).

The specific effect of IL-10 on NK-cell responses shows contradicting evidence. In a similar fashion to the role of IL-10 in T cells, several studies showed that IL-10 suppresses the inflammatory and cytotoxic responses in NK cells. The suppressive role of IL-10 on NK cells effector functions was demonstrated both directly and indirectly. Directly, T regulatory cells were shown to release IL-10 and as a result reduced NK cell cytotoxicity and IFN-γ expression (125). Later it has been demonstrated that IL-10 can downregulate NKG2D expression which mediates NK-cell effector functions (126). Indirectly, viral infections such as MCVM or LCMV were demonstrated to elevate IL-10 expression in myeloid cells like dendritic cells and monocytes (124, 127). As an example, in the experimental model of MCMV, IL-10 prevented NK-cell mediated licensing of dendritic cells, which resulted in reduced CD4 T cell activation and reduced viral clearance (127). Interestingly, NK cells exposure to IL-10 in combination with IL-15, mediated IL-10 release and as a result mediated infection persistence (128).

Surprisingly, IL-10 was also shown to assist in NK-cell functionality and survival during acute MCMV infection. In IL-10Rb deficient mice, reduced frequency of NK cells in the lungs and spleen was observed, which was attributed to elevated apoptosis. Here, IL-10 was shown to prevent NK cell apoptosis which is mediated by acute MCMV infection (129). Moreover, during human HCMV infection, a release of cmvIL‐10, a virokine homologous to human IL-10, was detected. NK cells stimulated with cmvIL‐10 were shown to have induced NK-cell cytotoxic responses and activation receptors expression. cmvIL‐10 was shown to mediate its effect with IL-10a receptor (130).

**Figure 4:**
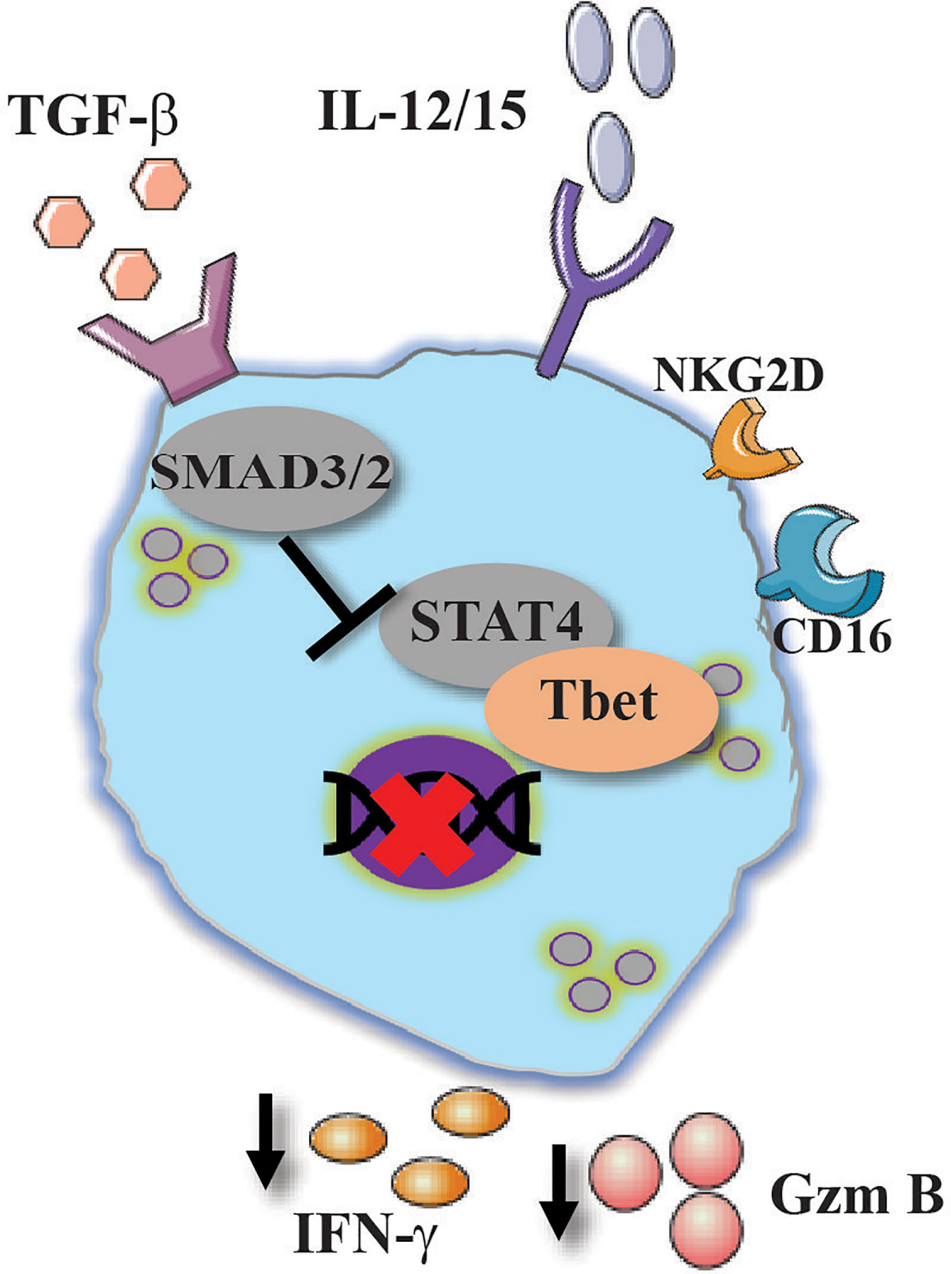
Conventional and unconventional effect of TGF-β on NK cells activation. TGF-β suppress NK cells activation by reducing the expression of CD16, NKG2D, IFN-γ and granzyme B via the canonical pathway by SMAD2/3.

**Figure 5:**
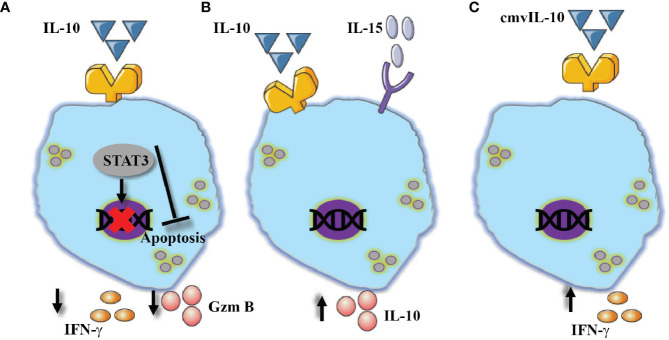
NK cells show diversified responses in the presence of IL-10. **(A)** Alone, reduced expression of IFN-γ and granzyme B, in addition to its ability to prevent apoptosis via STAT3 signaling pathway. **(B)** In combination with IL-15, increased expression of IL-10. **(C)** The virokine cmvIL-10 mediates NK cells activation which resulted in enhanced IFN-γ expression.

It appears that both TGF-β and IL-10 play a canonical role in suppressing NK-cell effector responses in order to protect the host from damaging inflammation. However, each of those cytokines was shown to have a non-canonical pathway in which they promote the survival and activation of NK cells. Further studies will clarify the intriguing role of both TGF-β and IL-10 regarding NK cells immune responses (**Figures 4** and **5**).

### Mixed Cytokine Milieus

The majority of pathologies are categorized as type 1, 2 or 3 diseases and patients are being treated according to the dominant phenotype of the disease (131–133). It appears that numerous pathologies which progress from an acute form, transition into a chronic stage and have the potential to aggravate and develop into subsequent pathologies, are characterized by a complex inflammatory milieu. For example, in COPD caused by patient’s smoking and additional environmental factors, the lung tissue is going through injury and remodeling, processes which are characterized by continuous release of IL-33 and IL-4 and TGF-β respectively (134–136). COPD patients suffer from malfunctioning epithelial tissue and thus, are more susceptible to microbial infections (137, 138). COPD exacerbation is mediated by respiratory microbial insults which modulate the release of cytokines such as IL-1β, IL-12 (139). COPD provides an example for a pathology which contains a mixed inflammatory environment.

**Figure 6:**
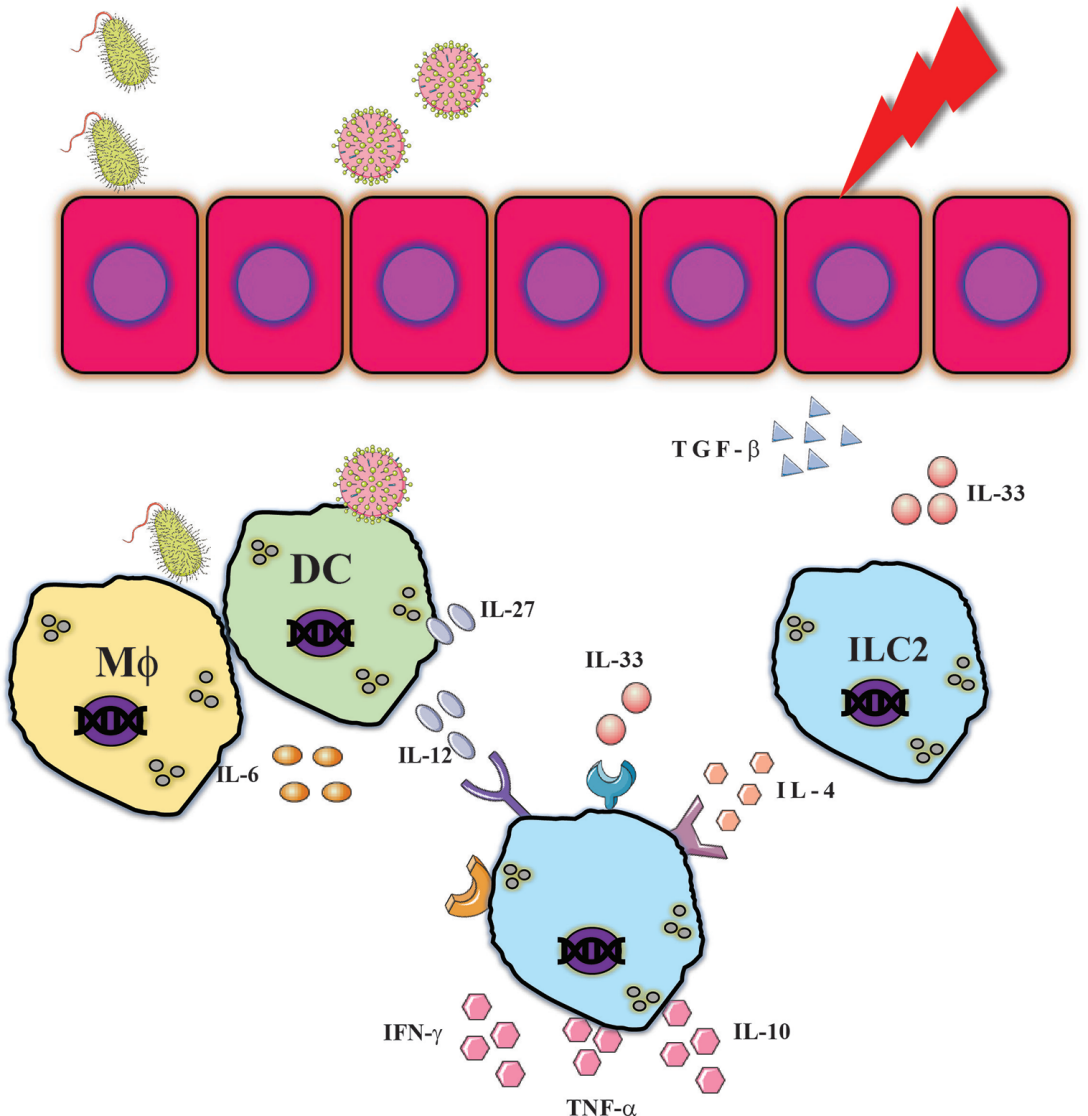
NK cells are exposed to complex inflammatory milieu. Various insults can provide innate immune responses which resulted in release of type 1 and type 2 cytokines which resulted in cytokines such as. IFN-γ, TNF-α or IL-10 which could assist in combating pathogens but can also mediate tissue injury.

Another example is atopic dermatitis (AD), which presents a diversity of phenotypes (140, 141). In atopic patients, the acute phase is mediated by type 2 cytokines such as IL-4 and IL-13 and leads to skin barrier impairment (141, 142). The modulation of the skin tissue by the acute phase of AD promotes tissue injury and mediates the release of IL-33 and microbes’ infiltration (e.g., *staphylococcus aureus*) resulting in release of type 1 cytokines such as IL-12 (143). Thus, the immune system of AD patients is exposed to a complex inflammatory milieu which dictates dysfunctional immune responses, suggested to lead to food allergy, asthma or chronic rhinitis (144). Indeed, in a subsequent disease to AD, asthma (145), which is characterized by mixed inflammatory milieu, NK cells demonstrated a dysregulated phenotype (146). NK cells derived from asthmatic compared to normal patients demonstrated reduced cytotoxicity while showing an increased expression of inflammatory cytokines. dysregulated phenotype, on one hand reduced cytotoxicity while expressing high levels of inflammatory cytokine, a phenomenon that might provide an explanation to the increased susceptibility of asthma patients to viral infection (147), and thus pose NK cells as having a pivotal role in mixed cytokines pathologies. The next section will bring several examples of how NK cells function in a complex inflammatory milieu (**Figure 6**).

NK cells were shown to have a crucial role in the clearance of RSV infection (148). A recent study highlighted the importance of NK cells in disease resolution in the presence of neutralizing antibodies and by performing ADCC (149). However, in the absence of neutralizing antibodies, RSV triggers NK cells inflammatory responses *via* IL-12, which in turn mediate the release of IFN-γ (150). Excessive expression of IFN-γ was shown to mediate lung injury and inhibit efficient clearance of RSV (151, 152). RSV was demonstrated to trigger the expression of both type 1 (e.g. IL-12) and type 2 (e.g. IL-4) cytokines (153). Enhanced expression of IL-4 in experimental RSV infection enhanced disease phenotype with increased IFN-γ expression but with reduced cytotoxicity (154).

The harmful effect of RSV is not limited to neonates, which lack the humoral response towards RSV. Studies have shown that RSV infection promotes the exacerbation of COPD and asthma (155, 156), which suggests that NK cells fail to perform efficient cytotoxic responses while maintaining high expression of IFN-γ and additional inflammatory cytokines, and thus contribute to tissue injury. However, the specific molecular mechanism which mediates those responses is in need to be clarified.

In contrast to RSV infection, Influenza infection was shown to be controlled on several levels; the activation receptors NKp46 and NKp44 were demonstrated to recognize viral hemagglutinin (HA) which results in viral neutralization (157). In addition, NK cells were shown to clear virally infected cells by cytotoxic responses. As previously mentioned, and as demonstrated by other studies, IL-27 meditates NK-cell viral clearance of influenza (158). In contrast, influenza infection in mice exposed to cigarette smoke, led to increased expression of IL-33 and hyper-activated the NK cells which contributed to excessive tissue damage (53). In addition, IL-4 was shown to reduce the cellular cytotoxicity in response to influenza infection (159), however additional studies are needed to elucidate the role of IL-4 on NK cell responses in the course of influenza infection.

Although the role of TGF-β during respiratory viral infection is suggested to be inhibitory by promoting viral evasion on one hand (160), while on the other hand impairing NK cells responses, an interesting beneficiary effect for the host was observed (161). In an experimental model of asthma, TGF-β was shown to downregulate NK-cell responses during influenza infection which resulted in reduced tissue injury (161). Additional studies should be conducted to examine the beneficial and harmful effects of TGF-β in the presence of respiratory viral infections, and at which time points post infection it can play each of the above-mentioned roles.

In summary, it appears that respiratory viral infections might take advantage of a situation of a mixed inflammatory milieu. In this type of environment, NK cells were shown to have reduced cytotoxic responses, however, when examined in the presence of non-type 1 cytokines, NK cells have the potential to increase the release of IFN-γ as well as additional cytokines, which in turn might enhance tissue damage or recovery from the disease.

## Concluding Remarks

Conventional rationale will suggest that type 2 cytokines (e.g., IL-33, IL-4) should counteract type 1 (e.g. IL-12) responses, and thus, NK cells should not be activated in the presence of type 2 cytokines. However, in the present review there are evidences which show unorthodox responses of NK cells to non-type 1 cytokines. In fact, in the majority of the discussed cases, NK cells were shown to express the NK-cell hallmark cytokine IFN-γ, in response to those cytokines alone, or in combination with type 1 cytokines such as IL-12 (11–13).

The common approach to research pathologies is to focus on one molecule, receptor, signaling pathway in one or several disease models. However, pathologies such as asthma, COPD, atopic dermatitis and even the 2020 pandemic COVID-19 are characterized by a heterogenous inflammatory environment which contains diversity of cytokines (143, 162–165), and dictates NK-cell immune responses. For example, according to the conservative cytokine response, NK-cell responses should be suppressed in conditions such as asthma or smoking, however, surprisingly it appears that in the presence of a mixed inflammatory environment NK cells fail to perform cytotoxic cell death, but remain fantastic cytokine producers, which results in exacerbated conditions (146).

A mixed inflammatory milieu is not limited to pathological conditions; at homeostasis, NK cells which are distributed in the blood as well as in tissues, are exposed to tissue remodeling, turnover and injury, all are mediated by the release of heterogenous cytokines which should be assumed to modulate NK cell responses. However, how NK cells function, express cytokines and which cellular or molecular mechanisms govern those responses, needs additional clarification.

Enhanced understanding of how complex inflammatory environments affect NK cells will assist us in developing and utilizing therapeutic approaches, which will modulate NK cells responses in a variety of pathologies in which NK cells play significant roles.

## Author Contributions

DO: Conceptualization, investigation, and writing—original draft. SNW: conceptualization, writing—original draft, and study supervision. All authors contributed to the article and approved the submitted version.

## Funding

This project was supported in part by Arnold W. Strauss Fellow Award (DO) and other support from the Cincinnati Children’s Research Foundation, a postdoctoral fellowship form American Heart Association (DO), and NIH awards DA038017, AI148080, AR073228, and AI145304.

## Conflict of Interest

The authors declare that the research was conducted in the absence of any commercial or financial relationships that could be construed as a potential conflict of interest.

## Abbreviations

ADAM, A disintegrin and metalloprotease; COPD, Chronic obstructive pulmonary disorder; GM-CSF, Granulocyte macrophage colony stimulating factor; IFN, Interferon; IL, Interleukin; ILC, innate lymphoid cells; MAPK, Mitogen-activated protein kinase; NK cells, Natural killer cells; STAT, Signal transducer and activator of transcription; RSV, Respiratory Syncytial Virus; TGF, Tumor transforming growth factor; TNF, Tumor Necrosis Factor; VEGF, Vascular endothelial growth factor.
